# High levels of LIGHT/TNFSF14 in patients with Prader–Willi syndrome

**DOI:** 10.1007/s40618-023-02050-2

**Published:** 2023-03-14

**Authors:** M. F. Faienza, G. Brunetti, D. Fintini, G. Grugni, M. G. Wasniewska, A. Crinò, G. D’Amato, L. Piacente, A. Oranger, M. Dicarlo, S. Colucci, M. Grano

**Affiliations:** 1 Department of Precision and Regenerative Medicine and Ionian Area, Section of Human Anatomy and Histology, University of Bari ‘A. Moro’, Bari, Italy; 2 Department of Biosciences, Biotechnologies and Environment, University of Bari Aldo Moro, Via Orabona, 4, 70125 Bari, Italy; 3 Endocrinology Unit, Pediatric University Department, Bambino Gesù Children’s Hospital, Rome, Italy; 4 Division of Auxology, Istituto Auxologico Italiano, Research Institute, Verbania, Italy; 5 Pediatric Unit, Department of Human Pathology in Adulthood and Childhood, University of Messina, Messina, Italy; 6 Reference Center for Prader-Willi Syndrome, Bambino Gesù Children’s Hospital, Research Institute, Rome, Italy; 7 Neonatal Intensive Care Unit, Di Venere Hospital, Bari, Italy; 8 Department of Translational Biosciences and Neurosciences, Section of Human Anatomy and Histology, University of Bari ‘A. Moro’, Bari, Italy

**Keywords:** Prader–Willi syndrome, LIGHT/TNFSF14, Bone disease, DXA

## Abstract

**Purpose/methods:**

Prader–Willi syndrome (PWS) is a rare genetic disorder displaying different clinical features, including obesity and bone impairment. LIGHT/TNFSF14 is a cytokine produced by immune cells affecting both fat and bone metabolism. The present study aimed to evaluate LIGHT serum levels in 28 children and 52 adult PWS patients compared to age and sex-matched controls, as well as correlations with parameters of bone and fat metabolism.

**Results:**

Median serum LIGHT levels were significantly increased in pediatric PWS with respect to controls [255.82 (284.43) pg/ml vs 168.11 (76.23) pg/ml, *p* ≤ 0.02] as well as in adult PWS compared to controls [296.85 (895.95) pg/ml vs 134.18 (141.18) pg/ml, *p* ≤ 0.001]. In pediatric PWS, LIGHT levels were positively correlated with weight-SDS, height-SDS, and glucose levels, and negatively with total 25 (OH) vitamin D, cholesterol, LDL cholesterol and triglycerides. Additionally, LIGHT levels were negatively correlated with total BMD and fat mass. In adult PWS, LIGHT levels were positively correlated with weight, HDL cholesterol and PTH, and negatively with glucose, insulin, HOMA-IR, total cholesterol, LDL cholesterol, triglycerides, calcium, phosphorus, 25(OH)Vitamin D as well as with instrumental parameters of bone and fat quality. Consistently, multiple regression analysis showed that LIGHT serum levels in pediatric and adult PWS were predicted by different parameters including 25 (OH) Vitamin D as well as DXA parameters of bone and fat quality.

**Conclusions:**

In PWS children and adults the high levels of LIGHT could represent a marker of the altered bone and fat metabolism.

## Introduction

Prader-Willi syndrome (PWS) is a rare complex genetic disorder, resulting from the inactivation of the Prader-Willi Critical Region on paternal chromosome 15 (q11-q13) which contains a cluster of imprinted genes [1]. The clinical features include neonatal hypotonia with initial failure to thrive, followed by hyperphagia and early childhood-onset obesity if not controlled, endocrine abnormalities, developmental delay, behavioral disorders, dysmorphic features as well as bone impairment [2]. In particular, PWS subjects display reduced bone mineral content (BMC) and total bone mineral density (BMD) [1, 3–5], as well as additional orthopedic problems and fracture risk [4, 6]. Moreover, in PWS subjects fat-free mass (FFM) is significantly lower than controls [7, 8], and muscle function as well as resting energy expenditure (REE) are impaired compared to subjects with common obesity, although REE results unaltered when adjusted for FFM [9]*.* Recently, it has also been reported that subcutaneous adipose tissue is a positive predictor for BMD in prepubertal PWS children [10].

Different mechanisms have been proposed to explain the bone impairment in PWS subjects, including the multiple endocrine abnormalities that requires treatment with recombinant growth hormone (rhGH) in childhood, and later sex steroid replacement therapy. Previously, we reported the involvement of RANKL, OPG, and sclerostin in the altered bone turnover of PWS subjects [5]. More recently, we also demonstrated the role of irisin, a molecule affecting bone and fat metabolism [11, 12] in PWS children and adults [13]. There is a network bridging the classic insulin target tissues (adipose tissue, muscle, and liver) and the cells of immune system that, in turn, release cytokines affecting/altering the homeostasis of these tissues [14]. Among the molecules produced by the immune cells, LIGHT/TNFSF14 (homologous to Lymphotoxins exhibiting Inducible expression and competing with herpes simplex virus Glycoprotein D for herpes virus entry mediator (HVEM), a receptor expressed by T lymphocytes) plays a role in modulating the crosstalk of the above-mentioned tissues.

A reduced bone mass has been demonstrated in *Tnfsf14* deficient mice [15], whereas high levels of this cytokine have been found in obese subjects [16, 17] and in some bone diseases [18–20]. However, the role of LIGHT in adipogenesis is still debated. Liu et al. showed that in vitro LIGHT sustains the adipogenic differentiation of human mesenchymal stem cells [21], whereas Tiller et al. reported that LIGHT inhibits adipogenesis without altering adipocyte metabolism [22]. Furthermore, there are no literature data on the involvement of this cytokine in syndromic obesity.

The aim of this study was to evaluate the serum levels of LIGHT in a cohort of children and adults affected with PWS. We also correlated the levels of this cytokine with anthropometric, metabolic and instrumental parameters of fat and bone.

## Materials and methods

### Patients

Eighty PWS subjects, 28 children (15 females, mean age 9.74 ± 3.79 years) and 52 adults (30 females, mean age 30.6 ± 10.7) were enrolled for this study. Forty-eight subjects had interstitial deletion of the proximal long arm of paternal chromosome 15 (del15q11-q13) (DEL15), while 32 patients had uniparental maternal disomy for chromosome 15 (UPD15). All PWS children were on rhGH treatment from at least 12 months (0.025–0.035 mg/kg/day). Furthermore, 6 out of the 52 PWS adults displayed growth hormone deficiency, consistent with a growth hormone (GH) response to GHRH plus arginine less than 4.2 ng/ml [23], and received rhGH therapy (0.23 mg/day). Additionally, 2 males and 10 females underwent sex steroid replacement treatment.

The controls included 26 children (12 females, mean age 9.36 ± 2.84 years), referred to the hospital for electrocardiographic screening or minor surgery, and 54 volunteers normal-weight adults (26 females, mean age 36.5 ± 12.5 years).

Six out of 28 PWS children (21%) and 26 out of 52 PWS adults (50%) were on vitamin D supplementation at the time of the study [cholecalciferol mean dosage: children 500 UI/daily (12.5 mcg/daily); adults 800 UI/daily (20 mcg/daily)].

Exclusion criteria for both patients and controls included the use of vitamin and mineral supplements, except for vitamin D, diagnosis of concurrent chronic diseases affecting bone metabolism (e.g., Cushing’s syndrome, hypothyroidism or hyperthyroidism, anorexia nervosa, celiac disease, etc.), the use of medications altering bone turnover, and fractures in the 6 months preceding the study. Written informed consent was signed from all the legal guardians, and from the patients when applicable. Local institutional review boards approved all procedures.

### Anthropometric measurements

All patients underwent anthropometric measurements (height in cm, weight in kg) and, for the pediatric age, data were expressed as standard deviation scores (SDS) [24]. Body mass index (BMI) was defined as weight in kilograms divided by the square of height in meters. The international standards for sex- and age-specific BMI percentiles were used for subjects aged 2–18 years [24]. BMI SDS was derived from the published Center for Disease Control and Prevention (CDC) standards [25]. The BMI cut-off point of > 2 SDS was used to define obesity, and between 1.4 and 2 SDS to define overweight for individuals < 18 years of age. Considering adult age, we considered as obese, overweight and normal-weight those subjects with a BMI > 30, in the range of 25–30 and < 25, respectively. The pubertal stages were assessed according to the Tanner criteria [26].

### Biochemical measurements

Blood samples were drawn under fasting conditions, centrifuged, and stored at −80 °C until required. Blood glucose, insulin, total cholesterol (TC), high (HDL) and low (LDL) density lipoprotein cholesterol, triglycerides (TG), were measured after overnight fasting in all subjects, using standard methods. Values of TC, LDL, HDL, and TG were considered in the normal range if within the 5th and the 95th percentile. Insulin resistance was assessed calculating the homeostasis model assessment (HOMA) [27]. Calcium (Ca) and phosphorus (P) concentrations were measured by the nephelometric method. Serum intact parathyroid hormone (PTH) and 25(OH) vitamin D were measured by immunological tests based on the principle of chemiluminescence using commercial kits (Liaison assay; DiaSorin, Stillwater, Minnesota, USA). Osteocalcin serum concentration was measured by enzyme immunoassay (IBL International GmbH, Hamburg, Germany). LIGHT levels were measured using a commercially available ELISA kit (R&D System srl, Minneapolis, MN, USA). For this assay the intra-assay coefficient of variability (CV) was % 4.6, whereas inter-assay CV was 4.3%. The minimum detectable dose of human LIGHT ranged from 1.2 to 16.5 pg/ml (mean 5.5 pg/ml), whereas the maximum levels is 2000 pg/ml. As quality control, we used a human LIGHT sample suggested by the kit manufacturer, commercially available.

### Dual X-ray absorptiometry (DXA)

Bone mass of the anterior–posterior lumbar spine (L1-L4), total body (TB) and total body less head (TBLH) in children was measured by DXA using a Hologic QDR Discovery, and the APEX-system software version 13.3 (Hologic Bedford, MA) with fan beam in array mode. The measurements were performed using standard positioning techniques. Quality control scans were performed daily using a simulated L1-L4 lumbar spine phantom. The lumbar spine DXA scan were analyzed to generate measures of L1-L4 vertebral areal BMD (LS-BMD, g/cm^2^), bone mineral content (LS-BMC, g), spine volumetric BMD (LS-BMAD, g/cm^3^), lumbar spine Z score (LS-BMD Z score, SDS) [28–30]. Bone variables were normalized for height to avoid any influence of growth on bone mass [30]. TB scans were obtained to estimate Fat Mass (FM, g), fat mass percentage (FM%), Fat Free Mass (FFM, g) and fat-free mass percentage (FFM%) expressed as percentage of TBLH.

### Statistical analyses

Sample size calculation is based on LIGHT level detected in previous studies on children [16] and adults [17]. A sample of 28 children and 52 adults will allow to detect a difference in LIGHT levels between PWS and controls with a level of significance equal to 0.05 and a study power of 0.8.

We used obese subjects as a reference group because they displayed bone impairment similar to PWS subjects, who can also be obese.

LIGHT levels, which are not normally distributed, are reported as median and interquartile. Other parameters are shown both as mean ± standard deviation and median with interquartile. The Kolmogorov–Smirnov test was used to evaluate the normality of parameter distribution. Mean values of parameters with normal distribution were compared using the Student *t* test, and correlations assessed with Pearson's correlation coefficient. Medians of parameters with a not normal distribution, were compared using the Mann–Whitney test while correlations were evaluated with Spearman's coefficient.

With regards to qualitative variables, in adult PWS association of rhGH therapy (yes/no/previous) and sex steroid therapy (yes/no/previous) were evaluated using the Kendall-tau coefficient (τB), whereas in pediatric and adult PWS for Vitamin D supplementation (yes/no), the correlation was calculated using the Point-Biserial correlation coefficient. Multiple linear regression analyses were used to evaluate the strength of association of each clinical and biochemical variable and LIGHT levels. In particular, for children, weight-SDS, Ca, P, 25 (OH) Vitamin D, and LS-Z score were used as independent variable to predict LIGHT levels. Differently, for adults, 25 (OH) Vitamin D, the different therapies, LS-Z-score, Fat Mass, osteocalcin, TB BMC, LS-BMC, LS-BMD and LS-BMAD were assayed to predict the cytokine levels. For the statistical analysis, the Statistical Package for the Social Sciences (SPSS) for Windows, version 26.0 (SPSS Inc., Chicago, IL, USA) was used. The results were considered statistically significant for *p* < 0.05.

## Results

### PWS pediatric population

**Fig. 1:**
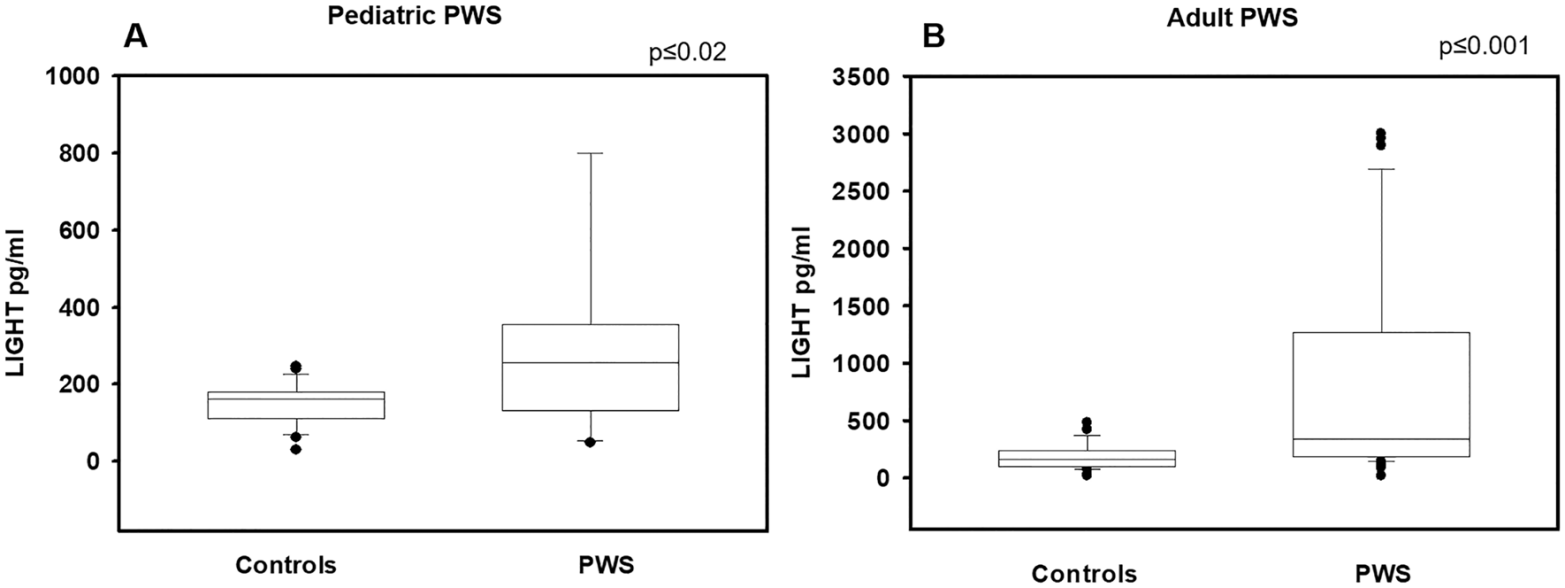
High levels of LIGHT in PWS patients. Median serum LIGHT levels are significantly increased in pediatric PWS (**A**) with respect to controls [255.82 (284.43) pg/ml vs 168.11 (76.23) pg/ml, *p* ≤ 0.02] as well as in adult PWS (**B**) compared with controls [296.85 (895.95) pg/ml vs 134.18 (141.18) pg/ml, *p* ≤ 0.001]

Table 1 shows the general and biochemical characteristics as well as instrumental parameters of fat and bone of PWS pediatric population. Median serum LIGHT levels were significantly increased in PWS children with respect to controls [255.82 (284.43) pg/ml vs 168.11 (76.23) pg/ml, *p* ≤ 0.02], Fig. 1A. LIGHT levels were positively correlated with weight-SDS, height-SDS, glucose levels, PTH and Vitamin D supplementation, and negatively with 25 (OH) vitamin D, total cholesterol, LDL cholesterol and triglycerides (Table 2A). As regard for DXA parameters, LIGHT levels were negatively correlated with total BMD, FM (g) and fat-free mass percentage (FM%) (Table 2A). Multiple linear regression analysis for LIGHT serum levels as dependent variable demonstrated that weight-SDS, Ca, P, 25(OH)-vitamin D levels and LS-BMD Z score were the most important predictors (Table 3).

**Table 1:** Clinical, biochemical and instrumental characteristics of study population

	Pediatric PWS *N* = 28	Pediatric controls *N* = 26	Adult PWS *N* = 52	Adult controls *N* = 54
Gender (male/female)	11/15	14/12	22/30	28/26
Age (year)	9.74 ± 3.79 *11.08 (6.63)*	9.36 ± 2.84 *9.42 (3.8)*	30.6 ± 10.7 *39 (16.07)*	36.5 ± 12.5 *37.00 (19.00)*
Tanner (I/II/III/IV/V)	23/3/2/0/0	22/2/2/0/0	0/0/25/25/2	0/0/0/0/54
Height SDS	− 0.27 ± 1.54 − *0.75 (2.03)*	0.36 ± 1.02 *0.45 (1.21)*	–	–
Weight SDS	1.40 ± 1.38 *1.15 (1.86)*	0.43 ± 0.87 *0.55 (0.93)*	–	–
BMI	25.38 ± 10.28 *27.83 (8.24)*	23.00 ± 1.2 *23.8 (0.87)*	35.4 ± 8.4^#^ *36.30 (12.40)*^*#*^	24.9 ± 1.9 *25.00 (2.5)*
BMI SDS	2.03 ± 1.76 *1.74 (1.48)*	0.25 ± 0.78 *0.29 (0.79)*	–	–
Laboratory parameters				
Total cholesterol (mg/dl)	169.5 ± 34.8 *159.87 (45)*	150 ± 20.1 *139.2 (19.2)*	176.5 ± 36.5 *182.00 (55)*	
LDL-C (mg/dl)	100.0 ± 30.0 *94.00 (27)*	80.2 ± 21.5 *79.2 (19.3)*	114.9 ± 33.2 *123.00 (43)*	
HDL-C (mg/dl)	55.04 ± 10.31 *54.00 (21)*	58.7 ± 8.9 *56.9 (9.1)*	49.9 ± 12.5 *49.00 (18)*	
Triglycerides (mg/dl)	83.76 ± 66.64 *64.00 (50)*	62.3 ± 18.6 *58.9 (15.8)*	96.7 ± 39.9 *9.50 (40)*	
Glucose (mg/dl)	79.29 ± 7.57 *78.00 (9)*	84.57 ± 10.2 *88.11 (10.42)*	89.4 ± 22.5 *91.00 (18)*	
Insulin (mU/l)	9.87 ± 4.37 *8.49 (6.71)*	9.31 ± 4.0 *9.24 (4.0)*	10.1 ± 3.9 *10.08 (6.31)*	
HOMA index	1.96 ± 0.97 *1.61 (1.32)*	1.69 ± 0.9 *1.62 (0.9)*	2.31 ± 1.20 *2.18 (1.93)*	
Osteocalcin (ng/ml)	85.44 ± 24.14* 95.00 (35.04)*	40.1 ± 18.1 *37.9 (18.9)*	21.76 ± 11.26 *19.00 (14.00)*	
PTH (pg/ml)	49.51 ± 18.87 *43.00 (34)*	20.23 ± 2.1 *19.3 (6.00)*	49.27 ± 17.85 *49.40 (34.00)*	
Ca (mg/dl)	9.82 ± 0.38 *9.80 (0.70)*	9.69 ± 0.38 *9.65 (0.50)*	9.53 ± 0.42 *9.50 (0.60)*	
P (mg/dl)	4.77 ± 0.60 *4.40 (1.00)*	4.49 ± 1.38 *4.51 (1.39)*	3.83 ± 0.44 *3.90 (0.70)*	
25 (OH) Vitamin D	22.38 ± 7.76 *23.20 (7.00)*		29.4 ± 11.2 *3.90 (0.70)*	
Fat parameters				
FM (g)	26,562 ± 7296 *23,760 (8390)*	–	39,940 ± 11,361 *42,703 (16,113)*	–
FM (%)	44.30 ± 6.44 *41.10 (12.70)*	–	48.03 ± 6.23 *49.30 (10.20)*	–
FFM (g)	32,311 ± 10,454 *28,910 (9360)*	–	39,860 ± 6033 *41,580 (8971)*	–
FFM (%)	53.41 ± 6.07 *55.90 (11.43)*	–	50.32 ± 5.94 *49.1 (9.56)*	–
FFM/FM	1.24 ± 0.31 *1.40 (0.60)*	–	1.08 ± 0.27 *1.20 (0.40)*	
Bone parameters				
LS-BMD Z score	0.08 ± 1.1 *0.59 (1.50)*		–	–
LS-BMD T-score			− 1.17 ± 1.06 − *1.13 (1.97)*	–
TBLH BMC gr	1323 ± 283 *1352 (379)*		2109 ± 287 *2742 (795.83)*	
TBLH BMD (g/cm^2^)	0.91 ± 0.09 *0.90 (0.073)*		1.14 ± 0.08 *1.32 (0.28)*	
LS-BMC (gr)	29.39 ± 10.10 *30.76 (8.97)*		52.40 ± 9.53 *68.13 (12.81)*	
LS-BMD (g/cm^2^)	0.71 ± 0.12 *0.70 (0.151)*		1.06 ± 0.14 *1.07 (0.077)*	
LS-BMAD (g/cm^3^)	0.14 ± 0.02 *0.14 (0.03)*		0.19 ± 0.03 *0.17 (0.001)*	

*PWS* Prader–Willi syndrome, *SDS* standard deviation score, *BMI* body mass index, *LDL-C* low-density lipoprotein cholesterol, *HDL-C* high-density lipoprotein cholesterol, *HOMA* *index* homeostasis model assessment index, *PTH* parathyroid hormone, *Ca* calcium, *P* phosphorus, *LS-* lumbar spine, *TBLH* total body less head, *BMD* bone mineral density, *BMAD* bone mineral apparent density, *BMD-Ht* height adjusted

*p < 0.05 pediatric PWS respect to pediatric controls

^#^*p* < 0.05 adult PWS respect to adult controls

Italic font reported data calculated as median and interquartiles

**Table 2:** A Correlation between LIGHT and other parameters for PWS children; B Correlation between LIGHT and other parameters for PWS adults

	LIGHT
A Correlation between LIGHT and other parameters for PWS children	
Weight SDS	0.222, *p* < 0.008
Height SDS	0.458, *p* < 0.0001
Glucose	0.179, *p* < 0.036
Total Cholesterol	− 0.501, *p* < 0.0001
LDL-C	− 0.296, *p* < 0.001
Triglycerides	− 0.253, *p* < 0.002
25(OH)Vitamin D	− 0.272, *p* < 0.001
PTH	0.339, *p* < 0.001
Vitamin D supplementation	0.347, *p* < 0.002*
Total BMD	− 0.733, *p* < 0.0001
FM (g)	− 0.733, *p* < 0.0001
FM (%)	− 0.622, *p* < 0.0001
FFM (g)	0.422, *p* < 0.025
B Correlation between LIGHT and other parameters for PWS adults	
Glucose	− 0.522, *p* < 0.0001
Insulin	− 0.257; *p* < 0.001
HOMA-IR	− 0.314; *p* < 0.0001
Total Cholesterol	− 0.164, *p* < 0.0001
LDL-C	− 0.271, *p* < 0.0001
HDL-C	0.084, *p* < 0.002
Triglycerides	− 0.270, *p* < 0.0001
Ca	− 0.199, *p* < 0.0001
P	− 0.163, *p* < 0.0001
25 (OH) Vitamin D	− 0.207, *p* < 0.0001
PTH	0.091, *p* < 0.001
Vitamin D supplementation	0.276, *p* < 0.0001
rhGH therapy	0.172; *p* < 0.0001*
Sex steroid therapy	− 0.293, *p* < 0.0001*
LS-Z score	− 0.115; *p* < 0.0001
TB BMC	− 0.105, *p* < 0.0001
LS-BMC	− 0.242, *p* < 0.0001
LS-BMD	− 0.294, *p* < 0.0001
LS-BMAD	− 0.234, *p* < 0.0001
FM (g)	− 0.195; *p* < 0.0001
FM (%)	− 0.224; *p* < 0.0001
FFM (%)	0.220; *p* < 0.0001

*Kendall-tau coefficient (τB)

**Table 3:** Multiple linear regression analysis for LIGHT in pediatric and adult PWS

Dependent variable	Independent variable	*β*	*p*	*r*
Model 1 (children)				
LIGHT			0.0001	0.955
	Weight SDS	− 0.289	0.0001	
	Ca	0.717	0.0001	
	P	2.2	0.0001	
	25 (OH) Vitamin D	− 1.776	0.199	
	LS-Z-score	0.147	0.318	
Model 2 (adults)				
LIGHT			0.0001	0.779
	25 (OH) Vitamin D	− 0.078	0.006	
	Sex steroid replacement treatment	− 0.189	0.0001	
	GH Therapy	0.561	0.0001	
	Vitamin D Therapy	0.201	0.0001	
	LS-Z-score	0.144	0.005	
	Fat Mass (FM)	0.222	0.0001	
	Osteocalcin	0.320	0.0001	
	TB BMC	0.172	0.0001	
	LS BMC	1.383	0.021	
	LS BMD	− 5.204	0.0001	
	L-BMAD	4.021	0.0001	

### PWS adults

Median serum LIGHT levels were significantly increased in adult PWS compared with controls [296.85 (895.95) pg/ml vs 134.18 (141.18) pg/ml, *p* ≤ 0.001], Fig. 1B. Additionally, in adult PWS LIGHT levels were positively correlated with weight, HDL cholesterol and PTH, and negatively with glucose, insulin, HOMA-IR, total cholesterol, LDL cholesterol, triglycerides, Ca, P and 25(OH)Vitamin D (Table 2B). LIGHT levels were also negatively related with TB BMC, LS-BMC, LS-BMD, LS-BMAD, FM (g), and positively with FFM%. Furthermore, LIGHT levels were positively correlated with vitamin D supplementation and rhGH therapy, but negatively related to sex hormonal replacement therapy. In adult PWS the best predictors for LIGHT levels were 25(OH)-vitamin D levels, sex steroid replacement treatment, rhGH therapy, vitamin D supplementation, LS-Z score, osteocalcin, TB BMC, LS-BMC, LS-BMD, and LS-BMAD (Table 3).

## Discussion

The present study demonstrated that both pediatric and adult PWS patients have higher serum levels of LIGHT compared with the controls. We also found interesting correlations between the serum levels of this cytokine and anthropometric parameters, bone markers and instrumental parameters of bone and fat quality. Previously, elevated LIGHT levels have been measured in some bone diseases, such as rheumatoid arthritis [31], osteolytic multiple myeloma [18], and bone metastatic non-small cell lung cancer (NSCLC) [20]. Furthermore, higher LIGHT levels have been reported in obese adults and children than controls [16, 17]. Consistently with the role of this cytokine in obesity, our results showed that LIGHT levels positively correlated with the weight-SDS in children and weight in adults, as well as with lipid metabolism in both the population. Using in vivo models, different studies tried to deepen the role of LIGHT in fat homeostasis. In detail, it has been reported that LIGHT decreases beige fat biogenesis [32] and it has a key role in the inflammatory responses of adipose tissue by increasing macrophage/T-cell infiltration and inflammatory cytokine expression [33]. Furthermore, by means of in vivo models mimicking obesity it has been found that high-fat diet (HFD) fed wild-type mice displayed high circulating levels of serum LIGHT, produced by liver and white adipose tissue. Interestingly, *Tnfsf14* deficient mice showed increased obesity, hepatosteatosis, insulin resistance, glucose intolerance, and mitochondrial dysfunction respect to wild-type mice on a HFD [34]. Additionally, in mice under high-fat high-cholesterol diet, *Tnfsf14* deficiency ameliorated glucose tolerance and insulin sensitivity, and it is also associated to decreased systemic inflammation and adipose tissue cytokine secretion [35]. Consistently with these literature data, our results showed the correlation of LIGHT levels with parameters of glucose homeostasis. Interestingly, we found an inverse correlation between LIGHT and glucose levels in adults. Literature data reported that PWS patients displayed a protected glucose metabolism, mainly if compared with obese controls [36]. A possible explanation of this finding has been related to the preferential accumulation of fat in PWS as subcutaneous adipose tissue [37], and larger amounts of this tissue can help to prevent fatty-acid induced insulin resistance [36]. Consistently, greater thigh subcutaneous fat mass may be related with better glucose metabolism in PWS subjects.

Additionally, the levels of this cytokine were inversely correlated with 25 (OH) vitamin D levels, suggesting the key role of vitamin D supplementation in these patients, as supported by the positive association that we found between LIGHT levels and Vitamin D supplementation. This represents an important issue due to the key role of Vitamin D on bone homeostasis and further sustained by findings in our previous report on PWS patients. In detail, we demonstrated that the irisin levels, an osteoanabolic myokine able to affect fat homeostasis, were reduced in PWS patients who were not supplemented with vitamin D [13].

Interestingly, in adult PWS subjects we observed a direct association between LIGHT and GH-therapy, whereas an inverse association between LIGHT levels and sex hormonal replacement treatment, thus sustaining the important role of GH and sexual steroid for bone health. As regard the instrumental bone parameters, LIGHT levels were inversely related with total BMD in pediatric PWS, whereas in adult PWS LIGHT levels were negatively related with LS-Z score, TB BMC, LS-BMC, LS-BMD and LS-BMAD. These instrumental results agree with the high levels of the cytokine in bone diseases. Similarly, previously we demonstrated the negative correlation of pro-resorptive cytokine RANKL with BMD [5]. In support of these data, previously we found that the increase of LIGHT levels is associated with an enhanced pro-osteoclastogenic capability in vitro [16, 18] as well as an increased percentage of circulating precursors [18] compared with the controls. In particular, in different diseases an enhancement of the pro-resorptive osteoclast number/activity is associated to altered BMD.

Furthermore, we also found that LIGHT serum levels were inversely related to FM (g), directly related with FFM (g) both in children and adults. This finding may be linked with the unusual body composition of PWS subjects, displaying low visceral adipose tissue and decreased muscle mass [37].

The strength of the study is that the enrollment and examination of patients were performed by a well trained and experienced teams from PWS centers of third level. A limitation is the multicenter recruitment of our subjects, which makes the interpretation of the data less reliable than those obtained in a single center. However, DXA instrument was identical in all centers and the assays used for biochemical measurements were the same in all laboratories. These latter belong to the Italian National Health System and are certified according to International Standards ISO 9001:2015 (www.iso9001.com), undergoing semi-annual periodic quality controls and inter-laboratory comparisons. Lastly, LIGHT level measurement was centralized. As additional limitation, it is important to underline that this is a cross-sectional observational study that informs us about possible associations/correlations and not causality.

Our study has both clinical and research implications. Regarding the clinical implications, the increased LIGHT levels could represent a hallmark of altered bone and fat homeostasis in PWS patients. Thus, the monitoring of the levels of this cytokine can help to have an overview of the health status of the patients, and consequently to quickly act to cure it. About the research implications, the findings of this paper are added to other literature data to sustain the role of LIGHT in bone and/or fat diseases, and thus the role of this cytokine in the regulation of bone/fat homeostasis. Therefore, both clinical and research implications highlighted that LIGHT could represent a good pharmacological target to improve bone/fat homeostasis thus counteracting obesity and bone disease in PWS subjects.

In conclusion, to the best of our knowledge this is the first study assessing LIGHT levels in PWS subjects. Overall, our results highlighted the elevated serum levels of LIGHT in pediatric and adult PWS patients, and its relationship with fat and bone impairment. Thus, LIGHT clinically may represent a marker of bone diseases, including PWS, and as advance for the research can further press on the development of molecules that may neutralize it in vivo.

## Data Availability

The datasets generated and analyzed during the current study are available from the corresponding author on reasonable request.
